# Mucosal immunity in upper and lower respiratory tract to MERS-CoV

**DOI:** 10.3389/fimmu.2024.1358885

**Published:** 2024-08-30

**Authors:** Khalid J. Shrwani, Waleed H. Mahallawi, Abdulrhman I. Mohana, Abdullah Algaissi, Nabil Dhayhi, Nouf J. Sharwani, Eyad Gadour, Saeed M. Aldossari, Hasan Asiri, Nader Kameli, Ayad Y. Asiri, Abdullah M. Asiri, Alaa J. Sherwani, Nigel Cunliffe, Qibo Zhang

**Affiliations:** ^1^ Department of Clinical Infection, Microbiology and Immunology, Institute of Infection, Veterinary and Ecological Sciences, University of Liverpool, Liverpool, United Kingdom; ^2^ Public Health Authority, Saudi Center for Disease Prevention and Control (SCDC), Jazan, Saudi Arabia; ^3^ Clinical Laboratory Sciences Department, College of Applied Medical Sciences, Taibah University, Madinah, Saudi Arabia; ^4^ Department of Antimicrobial Resistance, Public Health Authority, Riyadh, Saudi Arabia; ^5^ Department of Medical Laboratory Technology, College of Applied Medical Sciences, Jazan University, Jazan, Saudi Arabia; ^6^ Emerging and Endemic Infectious Diseases Research Unit, Health Sciences Research Center, Jazan University, Jazan, Saudi Arabia; ^7^ Department of Pediatrics, King Fahad Central Hospital, Ministry of Health, Gizan, Saudi Arabia; ^8^ Department of Surgery, Mohammed bin Nasser Hospital, Ministry of Health, Gizan, Saudi Arabia; ^9^ Department of Gastroenterology and Hepatology, King Abdulaziz National Guard Hospital, Ahsa, Saudi Arabia; ^10^ Department of Medicine, Faculty of Medicine, Zamzam University College, Khartoum, Sudan; ^11^ Medical Laboratory Technology Department, College of Applied Medical Sciences, King Saud University, Riyadh, Saudi Arabia; ^12^ Medical Laboratory Department, Prince Mohammed bin Abdulaziz Hospital, Riyadh, Saudi Arabia; ^13^ Department of Medical Laboratory Technology, Faculty of Applied Medical Sciences, Jazan University, Jazan, Saudi Arabia; ^14^ Intensive Care Unit Department, Al Inma Medical Group, Al Hayat National Hospital, Ministry of Health, Riyadh, Saudi Arabia; ^15^ Preventive Medicine Assistant Deputyship, Ministry of Health, Riyadh, Saudi Arabia; ^16^ Department of Pediatrics, Abu-Arish General Hospital, Ministry of Health, Gizan, Saudi Arabia; ^17^ Academic and Research Departments, Section of Immunology, School of Biosciences, University of Surrey, Surrey, United Kingdom

**Keywords:** MERS-CoV, immune cells, mucosal, lung, tonsils, cytokines, chemokines, upper and lower respiratory tracts

## Abstract

**Introduction:**

Middle East respiratory syndrome coronavirus (MERS-CoV) has emerged as a deadly pathogen with a mortality rate of up to 36.2%. MERS-CoV can cause severe respiratory tract disease and multiorgan failure. Therefore, therapeutic vaccines are urgently needed. This intensive review explores the human immune responses and their immunological mechanisms during MERS-CoV infection in the mucosa of the upper and lower respiratory tracts (URT and LRT, respectively).

**Objective:**

The aim of this study is to provide a valuable, informative, and critical summary of the protective immune mechanisms against MERS-CoV infection in the URT/LRT for the purpose of preventing and controlling MERS-CoV disease and designing effective therapeutic vaccines.

**Methods:**

In this review, we focus on the immune potential of the respiratory tract following MERS-CoV infection. We searched PubMed, Embase, Web of Science, Cochrane, Scopus, and Google Scholar using the following terms: “MERS-CoV”, “B cells”, “T cells”, “cytokines”, “chemokines”, “cytotoxic”, and “upper and lower respiratory tracts”.

**Results:**

We found and included 152 studies in this review. We report that the cellular innate immune response, including macrophages, dendritic cells, and natural killer cells, produces antiviral substances such as interferons and interleukins to prevent the virus from spreading. In the adaptive and humoral immune responses, CD4^+^ helper T cells, CD8^+^ cytotoxic T cells, B cells, and plasma cells protect against MERS-CoV infection in URT and LRT.

**Conclusion:**

The human nasopharynx-associated lymphoid tissue (NALT) and bronchus-associated lymphoid tissue (BALT) could successfully limit the spread of several respiratory pathogens. However, in the case of MERS-CoV infection, limited research has been conducted in humans with regard to immunopathogenesis and mucosal immune responses due to the lack of relevant tissues. A better understanding of the immune mechanisms of the URT and LRT is vital for the design and development of effective MERS-CoV vaccines.

## 1 Introduction

Coronaviruses (CoVs) pose a serious global health threat to humans and animals. Four different genera of CoVs have been described: alphacoronaviruses (αCoVs), betacoronaviruses (βCoVs), gammacoronaviruses (γCoVs), and deltacoronaviruses (δCoVs) (1). Currently, seven main CoVs are known to infect humans: HCoV-NL63, HCoV-OC43, HCoV-HKU1, HCoV-229E, severe acute respiratory syndrome coronaviruses (SARS-CoV and SARS-CoV-2), and Middle East respiratory syndrome coronavirus (MERS-CoV) (2, 3). The hCoV strains cause the common cold and infect only the upper respiratory tract (URT), while SARS-CoV, SARS-CoV-2, and MERS-CoV also infect the lower respiratory tract (LRT), leading to severe complications, including death (4). MERS-CoV was first isolated in 2012 from the saliva of an elderly Saudi patient with severe acute pneumonia and renal failure (5). Since 2012, it has been detected in 27 countries, resulting in 2,622 laboratory-confirmed cases, including 950 deaths, with a mortality rate of up to 36.2% (1–5). MERS-CoV is a zoonotic pathogen that has several potential mechanisms of transmission (6). Transmission is likely to occur directly via contact with any infected patients or animals and indirectly, such as contact with camel waste through cleaning camel corrals or the consumption or use of camel urine, unpasteurized milks, and raw meats. Indirect transmission of MERS-CoV can also occur through contact with contaminated surfaces in hospitals, laboratory, public places, or homes (7). MERS-CoV primarily infects the respiratory tract in humans, predominantly infecting and replicating in the respiratory epithelium in the URT and LRT (8).

An effective MERS-CoV vaccine should protect humans and animals and prompt a long-lasting immune response, characterized by neutralizing antibodies (Nabs) and cellular immunity. Several experimental MERS-CoV vaccines are in development. The chimpanzee adenovirus developed at Oxford University, vector version 1 MERS-CoV (ChAdOx1-MERS-CoV) vaccine, is the furthest advanced, and a phase 1b trial has been successfully completed in healthy Middle Eastern adults (9–11).

Currently, no licensed MERS-CoV vaccines exist, representing a matter of great public health concern. The understanding of the acquired immune responses and their underlying mechanisms will facilitate the design of safe and effective vaccine platforms. Additionally, limited information is available on the T- and B-cell response and mucosal cytokine and chemokine production during MERS-CoV infection. This extensive review aims to provide a critical summary of potential protective mechanisms and the immunity elicited in the URT and LRT to assist in the design of a safe, effective, and protective vaccine regimen.

## 2 Structure of MERS-CoV

**Figure 1:**
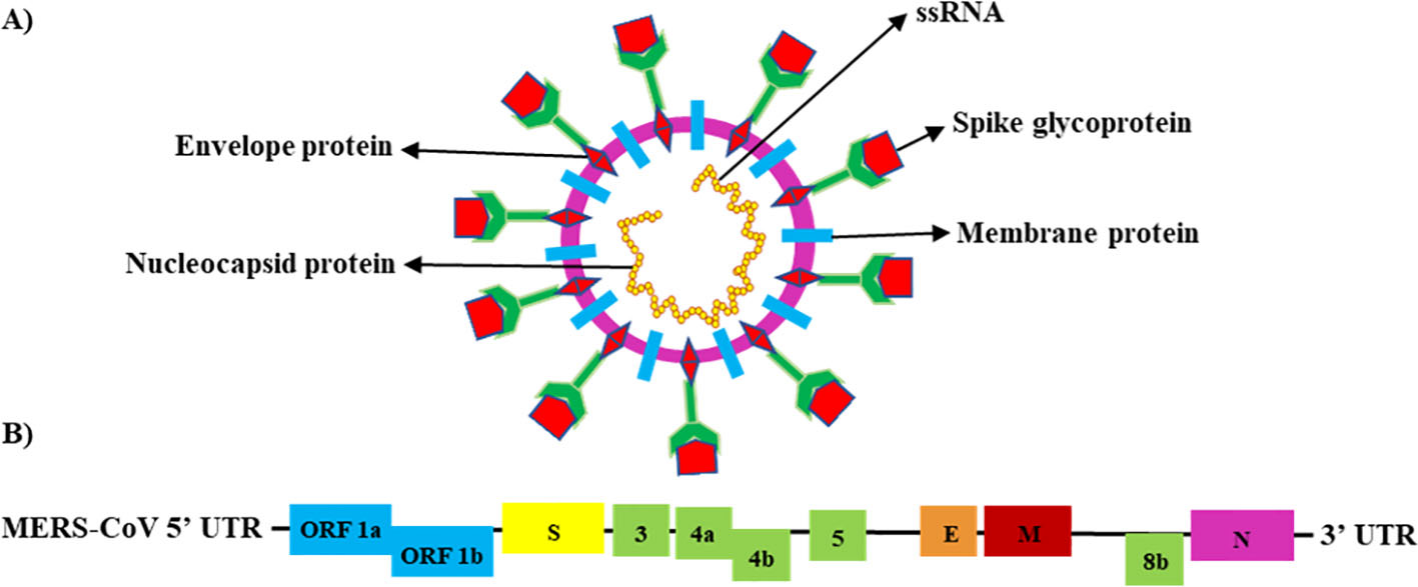
Schematic structure of the MERS-CoV genome (18). **(A)** Schematic structure of the MERS-CoV virion (ssRNA) and its major structural proteins; N protein, M protein, E protein, and S glycoprotein. **(B)** MERS-CoV genomic structure, with the untranslated region (UTR) 5′ and 3′; open reading frame regions ORF1a, ORF1b, ORF3, ORF4a, ORF4b, ORF5, and ORF8; S glycoprotein; E protein; M protein; and N protein.

MERS-CoV is classified as a Group IV virus based on the Baltimore Classification System (BCS) (12). It is a positive-sense, linear single-stranded RNA (ssRNA) virus with an enveloped genome ranging from 26 to 32 kilobases. The virus particle is spherical and symmetric, ranging from 77 to 131 nm in size. It contains at least 11 predicted open reading frames (ORFs; ORFs-1a, -1b, -S, -3, -4a, -4b, -5, -E, -M, -8b, and -N) and 16 functional non-structural proteins (NSPs) (kb) (13, 14). The MERS-CoV genome encodes for four structural proteins, the nucleocapsid (N) protein, membrane (M) protein, envelope (E) protein, and spike (S) glycoprotein, and five accessory proteins (15–17) (**Figure 1**).

The life cycle of MERS-CoV begins with the binding of the S glycoprotein to dipeptidyl peptidase-4 (DPP4), the host cell receptor, through the receptor-binding domain (RBD) on the S1 subunit of the S glycoprotein (19). This binding triggers a conformational change in the S2 subunit, which results in a close association of the viral particle and the cell membrane, facilitating membrane fusion and virus entry into the cytoplasm (20). Following the fusion of viral particles and cellular membranes, MERS-CoV RNA genomes are disassembled inside the target cells, releasing the N protein and genetic materials into the cytoplasm after the translation of ORF-1a and ORF-1b to pp1a and pp1ab, respectively, and the replication of genomic RNA. The replicated viral genome, N, and genomic RNA are assembled to form a helical N, which then cooperates with the S glycoprotein, M protein, and E protein to produce an aggregated virion (21). The viral structural proteins S, M, and E are then translated and use the secretory pathway system to transfer proteins across the endoplasmic reticulum–Golgi intermediate compartment (ERGIC) (22). N protein encapsulates viral genomes to form a mature virion after budding into the membranes of the ERGIC. Following assembly, the budded vesicles containing mature virions are transported to the host cell surface and released (23, 24). After releasing the new MERS-CoV progeny, the host innate immunity utilizes multiple strategies to detect and target viral particles (25, 26).

## 3 The host innate immunity in response to MERS-CoV infections

**Figure 2:**
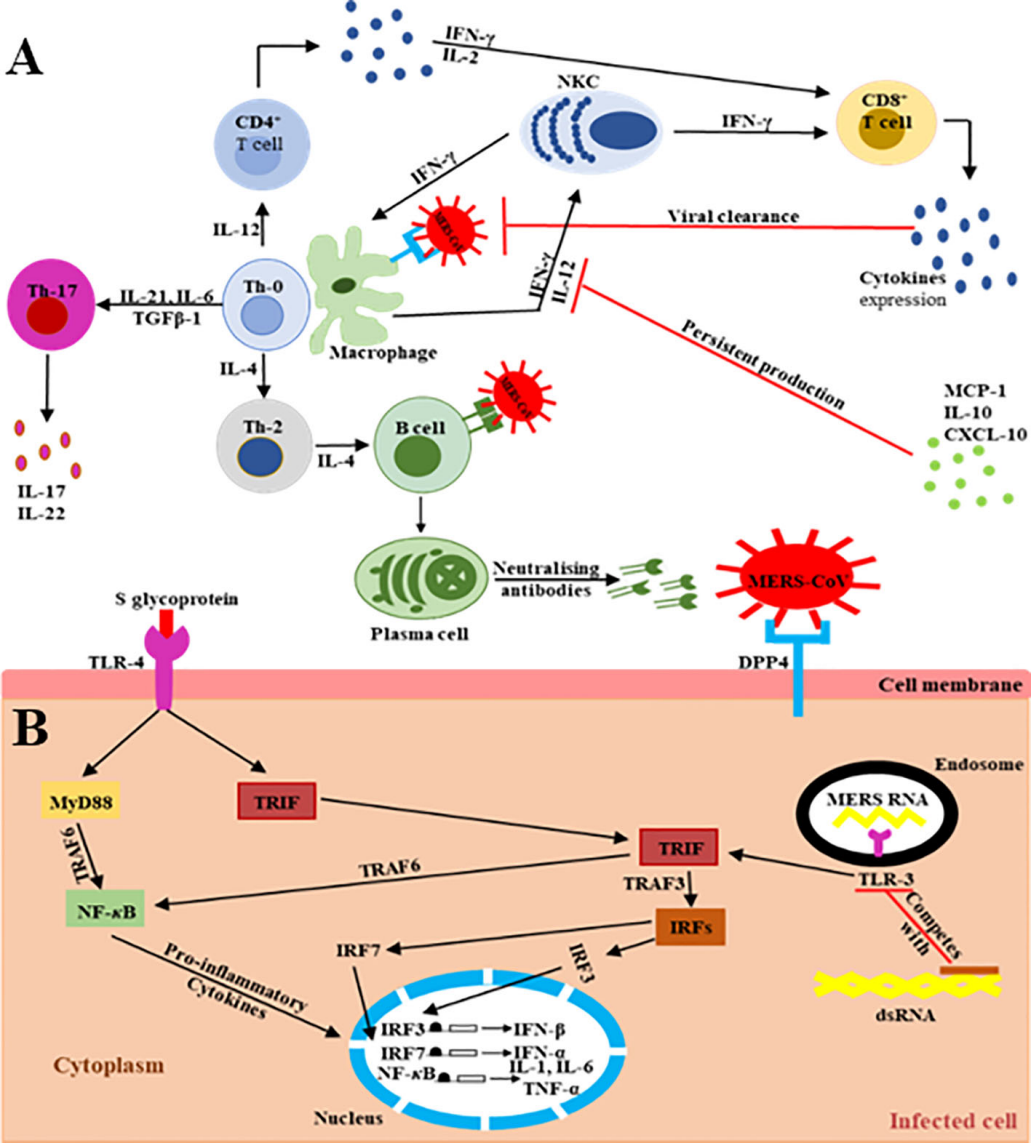
Schematic representation of the extra/intracellular immune response to MERS-CoV infection (34). The communications (interactions) between invading viruses and host cells lead to the generation and release of robust immune effective mediators. **(A)** Extracellularly, MERS-CoV binds its target DPP4 receptors on the cell membrane of the target cells, leading to the presence of genomic materials in the Th-0 cells. Consequently, the CD4^+^ T lymphocytes are activated and differentiate into Th-1, Th-2, and Th-17 subsets that secrete high amounts of subset-specific cytokines valuable in immune response enhancement. The cytotoxic effect of CD8^+^ T cells for the clearance of MERS-CoV is supported by the production of IFN-γ and the cytolytic granules containing mainly perforin and granzyme B. Plasma cells are responsible for the production of MERS-CoV-specific antibodies, some of them being able to neutralize the virus, stopping its dissemination. The duration of virus-specific antibody responses is not yet known. The binding of MERS-CoV S protein to DPP4 receptors on the target host cells causes the release of virus genomic RNA in the cell cytoplasm. **(B)** Intracellularly, host immune responses to double-stranded ribonucleic acid (ds-RNA) can be partially produced during viral replication. The presence of ds-RNA activates the endosomal TLR-3, which prompts the signaling pathways to activate NF-*κ*B via TRAF-6 and IRFs via TRAF-3 to produce both *IFN*-I and pro-inflammatory cytokine markers. The production and activation of *IFN*-I plays a key role by enhancing and releasing specific antiviral proteins to warn and protect un-infected cells during MERS-CoV infection. The activation of pro-inflammatory cytokines through the MyD-88-dependent signaling pathway may occur via the interactions between the TLR-4 and MERS-CoV S protein. The production and secretion of large amounts of cytokines and chemokines, such as IP-10, IL-10, and MCP-1, are stimulated to intracellularly target the MERS-CoV infection. Consequently, lymphocytes and leukocytes are recruited by these cytokine and chemokine markers to the site of infection to clear the virus.

The main MERS-CoV receptor, DPP-4, is broadly expressed on human URT/LRT cells, including submucosa glands, nasal passages, pharynx, and sinuses, as well as in lungs, bronchi, bronchioles, and alveoli (19–29). Therefore, the host innate immune response at the site of infection is directly triggered during virus particle attachment, mainly through the S glycoprotein to its target cell receptors and entry into the appropriate cell. The primary effector cells of innate immunity are mainly antigen-presenting cells (APCs), such as macrophages and dendritic cells (DCs), which identify the virus components (27–29). The pattern recognition receptors (PRRs) in APCs play a key role and act as crucial mediators of host innate immunity, capable of identifying the pathogen-associated molecular pattern receptors (PAMPs) initiated by viral replication, which result in the rapid endorsement of distinct antiviral signaling pathways in response to the invasive infection (30–32). They are vital in recognition of structural components of MERS-CoV RNA particles (33). TLRs recruit two different adaptor molecules at the site of viral detection: toll/interleukin-1 receptor (TIR-1) domain-containing adapter-inducing interferon-β (TRIF) and myeloid differentiation primary response-88 (MyD-88). These intracellular adaptor proteins play a vital role in activating the mitogen-activated protein kinase (MAPK) and NF-*κ*B pathways responsible for enhancing the production of pro-inflammatory cytokine markers (**Figure 2**) (32, 35–40).

Type II IFNs (IFN*-*II), chiefly interferon-gamma (IFN-γ), also possess specific antiviral activity by signaling through the JAK/STAT pathway via the release of specific major histocompatibility complex (MHC) proteins to block virus distribution (41, 42). IFN-γ plays a critical role by stimulating CD8^+^ cytotoxic T cells and natural killer cells (NKCs) that rapidly eliminate virus particles (43). According to Mahallawi et al. (44), IFN-γ levels are significantly higher in MERS-CoV-infected patients than in healthy individuals. Thus, elevated IFN-γ is important for eliciting immune responses against MERS-CoV and preventing severe illness and death (45, 46). DCs and their cytokine expressions also play a key role in enhancing immunity via activating T cells after migrating from the peripheral tissues to the lymphoid tissue (47). Therefore, it is vital to fully understand the signaling of IFN-γ because of its significant implications in host immunity.

## 4 The host adaptive immunity in response to MERS-CoV infection

### 4.1 T cell-mediated immune response to MERS-CoV

T cells are key essential immune factors for targeting and clearing viral infections. Following MERS-CoV infection, antigens are processed and presented to immune effector cells, chiefly helper (CD4^+^) T cells, facilitating virus-specific antibody production. T helper cells can also activate the antigen-binding B cells to differentiate into antibody-secreting plasma cells. This process is referred to as T-dependent B-cell activation (34, 48–50).

Virus-specific effector CD8^+^ cytotoxic T cells also produce high levels of immune effector cytokines, such as IFN-γ, TNF-α, and IL-2; chemokines, such as chemokine (C-X-C motif) ligand-9 and -10 (CXCL-9 and -10); and cytotoxic granules, mainly perforin and granzyme B, after the virus peptide is recognized on target cells at the site of infection (51–54). Ying et al. (50) and Manni et al. (51) found that CD4^+^ T-helper and CD8^+^ cytotoxic T cells are important immune cells in fighting MERS-CoV infection.

### 4.2 B cell-mediated immune response to MERS-CoV

Following MERS-CoV infection, defensive and lasting immune responses to viral infections typically result from a combination of immune effector cells: B cells after their differentiation into plasmablasts and plasma cells and their subsequent production of specific and Nabs and T cells that are responsible for cellular immunity and for supporting the humoral immune responses. Some antibodies neutralize the virus and prevent viral infectivity, which is critical in preventing virus entry into target host cells (6, 34).

MERS-CoV-specific antibody responses have been detected between post-infection days 14 and 21. Choe et al. (55) and Park et al. (56) found robust anti-MERS-CoV antibody responses persisting for over 1 year in all MERS-CoV survivors who experienced severe clinical symptoms. This finding was also supported by Alshukairi et al. (57), who found increasing anti-MERS-CoV antibody concentrations among recovered healthcare workers over 18 months after infection. The persistence of their antibody responses was also related to disease severity. Anti-SARS-CoV-specific antibody responses in SARS-CoV survivors have been shown in another study to persist for approximately 2 years post-infection before slowly reducing and disappearing entirely by 6 years post-infection (58, 59).

While anti-MERS-CoV-specific antibody responses can persist for at least 2 years, such responses among survivors of MERS disease have been found in numerous studies to be lower and short-term in asymptomatic or mildly ill patients than those in severely ill patients (60–63).

## 5 Immune mechanisms in the URT (mucosal immunity, e.g., tonsil mucosal immune responses)

### 5.1 Innate immune responses in the URT

The URT epithelial cells of the airway are considered the first line of defense against invasive viral infection, providing a mucociliary escalator mechanical barrier that uses mucus, cilia, and coughing mechanisms to expel particles and infectious microorganisms (64, 65). This mechanical barrier consists of DCs, tissue-resident macrophages, and URT epithelium cells. The DCs and tissue-resident macrophages express PRRs that recognize viral particles presented by receptors, like PAMPs and damage- or danger-associated molecular patterns (DAMPs) (66). The recognition of the virus leads to the recruitment of effector immune cells to the infection site, resulting in controlling the spreading of the virus or killing it.

The nasopharynx-associated lymphoid tissue (NALT), known as the mucosal immune component in the URT, enhances the production of a wide range of immune effector cells against several respiratory pathogens, and the nasal cavity has long been recognized as an immune barrier in bony vertebrates. Mucosal immune responses are generally regulated via myeloid cells (MCs) with specific functions to control infectious pathogens, comprising macrophages, monocyte-derived dendritic cells (moDCs), conventional dendritic cells (cDCs), and plasmacytoid dendritic cells (pDCs) (67, 68). The NALT plays crucial roles in the production of the innate and acquired immune responses at the mucosal site, accelerating the induction of Th-1 and Th-2 polarized lymphocytes and stimulating a high rate of immunoglobulin A secretion by immunoglobulin A (IgA)*-*committed B cells (67, 69). Tonsillar cells express various cytokines with antiviral activities, such as TNF-α, IFN-α, IFN-β, IFN-γ, transforming growth factor β-1 (TGF β-1), IL-1β, IL-6, IL-17, IL-28, and IL-29, in response to viral infection (70).

### 5.2 Adaptive immune responses in the URT

Tonsillar T cells are essential for the defense against MERS-CoV infections in the URT; CD4^+^ T cells facilitate the production of virus-specific antibodies by activating B cells in a T-dependent manner, while cytotoxic CD8^+^ T cells are killing MERS-CoV-infected cells and interact with the humoral antibody for eliminating the initial infection (71–77).

Tonsillar IgG, secreted by plasma cells, is the predominant antibody in the nasopharyngeal and palatine tissues (71, 72). Scadding (71), Wohlford et al. (72), and Boyaka et al. (73) found that the tonsils contained up to 10^9^ lymphoid cells, of which over 60% were B cells and up to 50% were T cells. CD4^+^ T cells are the largest subset, comprising approximately 80% of the total T-cell population (71–73).

IgA antibody is abundant in the saliva, respiratory secretions, and mucosal glands (74). It plays a crucial role in preventing respiratory viral infections and stimulating mucosal immunity. Secretory IgA (SIgA) protects mucosal surfaces by directly cross-linking viral particles (S glycoprotein) to prevent their contact with the surface of epithelial cells and facilitating their elimination by peristalsis or mucociliary movement, a mechanism known as immune exclusion (75). The induced antiviral mucosal antibody responses, consisting mainly of the SIgA isotype, play a direct role in antibody-mediated interactions with the receptors for the virus, preventing its attachment in this manner (76, 77). Recent studies have revealed that SIgA can stimulate monocyte chemoattractant protein-1 (MCP-1), granulocyte-macrophage colony-stimulating factor (GM-CSF), IL-6, and IL-8 production via normal human lung fibroblasts (NHLFs). SIgA and IgG antibodies may also promote antibody-dependent cellular cytotoxicity (ADCC) by synergistically enhancing mucosal immunity to accelerate the process of eradicating the invasive respiratory viral infection (78–80).

## 6 Immune system mechanisms in combatting MERS-CoV in the LRT (lungs)

### 6.1 Innate immune responses in the lungs

Respiratory tract cells are constantly exposed to external pathogens, making the lungs the most susceptible site for microbial infection. MERS-CoV can commonly infect and spread into the bronchi and bronchioles, resulting in cellular variations and localized tissue inflammation, similar to URT infections (81). Almost all human cells use PRRs to locate viruses by identifying the PAMPs or molecules related to the infectious agents (81). MERS-CoV infection triggers and activates PRRs expressed on DCs, macrophages, and respiratory epithelial cells. This activation facilitates the production and release of IFNs-I, IFNs-II, IFNs-III, and other pro-inflammatory mediators, such as cytokines, chemokines, and antiviral products, which prompt the host’s defense mechanism by initiating host innate and adaptive immunity (81). The innate lymphoid cells (ILCs) are considered innate immune cells that reside in both lymphoid and non-lymphoid mucosal tissues, and all three subsets (ILC1, ILC2, and ILC3) play a protective role in respiratory infections (82). Specifically, ILCs are the first line of defense important for blocking respiratory infection (82).

IFNs-I (for instance, IFN-α and IFN-β) play a key role in enhancing the functional activities of the lymphocytes and directly induce the production and release of IFNs-II, chiefly IFN-γ, which motivates the macrophages and phagocytosis processes and promotes antigen presentation by DCs (83, 84). IFNs-I also activate the CD8^+^ T cells and NKCs, hence destroying the infected cells and inhibiting viral spread (83). Generally, IFNs fulfill the critical function of directly and indirectly stimulating the humoral cells through T-cell and DC activation and endorse a strong, potent cellular and humoral immune response (81, 84). IFNs-III, such as interferon-lambda1 (IFN-λ1) [interleukin-28A (IL-28A)], interferon-lambda2 (IFN-λ1) [interleukin-28B (IL-28B)], and interferon-lambda3 (IFN-λ1) [interleukin-29 (IL-29)], activate the JAK/STAT signaling pathway and thereby provoke intrinsic and extrinsic antiviral immunological markers in human airway epithelium cells. The more IFNs-III are secreted by the respiratory epithelia cells, the more likely they are to combat respiratory viral infection (81, 83–85). Generally, IFNs are widespread throughout the human body and positively affect the host immune responses in addition to playing the critical functions of directly and indirectly stimulating the humoral cells through T-cell and DC activation and endorse a strong, potent cellular and humoral immune response (81, 83–85). However, they might inadvertently increase lung inflammation and damage, particularly through acute and severe virus infection (81, 83–85).

Following MERS-CoV infection, *airway epithelial cells* release a wide variety of host cytokines beyond the IFNs that are formed by the interior airway epithelium tissues, including GM-CSF, the granulocyte colony-stimulating factor (G-CSF), TNF-α, and IL-6. GM-CSF and G-CSF stimulate the differentiation and production of MC lineage (86). TNF-α stimulates cytokine production and cytotoxic activity to diminish and impair viral replication by activating the leukocytes and endothelial cells (87). IL-6 assists innate immunity to become adaptive immunity through reducing the activity of the neutrophils, alongside enhancing the recruitment, production, proliferation, differentiation, and activation of the T cells and monocytes (88). GM-CSF elicits the activation, proliferation, and differentiation of T and B cells and of monocytes (89).

*Respiratory epithelium cells* release various antiviral and antimicrobial products that prevent MERS-CoV infection. Antiviral products, such as lactoferrin, secretory leukocyte proteinase inhibitor (SLPI), and lysozyme, are abundant in the mucosal epithelial tissues. These tissues also produce several chemokine molecules that induce the migration of innate and adaptive immune cells to the infection site in the lungs (90). In the lungs, the IL-8/chemokine (C-X-C motif) ligand-8 (CXCL-8) recruits neutrophils and enhances degranulation, which leads to the secretion of pro-inflammatory and cytotoxic mediators that may increase the life span of the neutrophils. The production of those pro-inflammatory markers prompts the migration of innate and adaptive immune cells to the infection site for controlling MERS-CoV infection.

IP-10/chemokine (C-X-C motif) ligand-10 (CXCL-10) is another inflammatory marker that can activate the chemotaxis of NKCs, monocytes, DCs, and T cells in coordination with several cytokine molecules. In addition to conferring protection against respiratory viral infection, it contributes to the recruitment of leukocyte cells, initiates a heightened inflammatory response, and induces dysfunctional immune-mediated lung damage (91).

Chemokine (C-C motif) ligand-5 (CCL-5), also called Regulated upon Activation, Normal T-cell Expressed, and Secreted (RANTES), plays a crucial role in recruiting and activating several cell types expressing CCR-5, such as monocytes, macrophages, neutrophils, DCs, T, B, and NK cells that could prevent and control MERS-CoV infection, as reported for *ex vivo* MERS-CoV-infected human lung tissues (92–94). Similarly, mice infected with respiratory syncytial virus (RSV) also displayed protective immune responses in the lungs (93).

*Alveolar epithelial cells* (AECs) and *broncho-alveolar cells* (BACs) produce and release four surfactant proteins, SP-A, SP-B, SP-C, and SP-D. SP-A and SP-D function as secreted collectins and soluble PRRs that identify several viral and microbial PAMPs and enhance pathogens’ opsonization (81). Collectins stimulate neutrophils and macrophages and can facilitate viral detection, phagocytosis, and clearance (81). The release of those proteins plays key roles in the immune defense by recruiting immature DCs, immune effectors, and memory cells; enhancing cytokine and chemokine production; promoting pro-inflammatory immune responses; and disrupting the envelopes of invasive viruses such as MERS-CoV in addition to neutralizing their particles (81, 94–96).

*Endothelial cells* play a critical role in regulating immune responses, predominantly primary innate immune responses against invading viruses, and facilitating the migration of the leukocytes, leading to the release of various pro-inflammatory cytokines and chemokines, including IFN-α, IFN-β, IFN-γ, IL-6, TNF-α, monokine induced by gamma (MIG)/CXCL-9, IP-10/CXCL-10, and MCP-1/chemokine nomenclature C-C motif chemokine ligand-2 (CCL-2) (97, 98). The expression of those pro-inflammatory cytokines and chemokines plays an important function of signaling pathways and regulating the movement and localization of effector immune cells at the site of infection for destroying MERS-CoV.

*Respiratory alveolar macrophages* (AM) and *interstitial macrophages* (IM) directly respond to pathogens in the lower airways due to their unique position within the airspaces in the alveoli. Following the encounter with an invasive virus, such as MERS-CoV, the lung alveolar macrophages are responsible for early cytokine release and IFN production, besides their role in modulating and regulating secretion of pro-inflammatory markers helping to initiate immune responses able to annihilate the virus (85, 94). These cells can also inhibit unsuitable inflammatory immune responses during MERS-CoV infection as they regularly encounter harmless pathogens (90).

*Lung neutrophils* also contribute to eradicating MERS-CoV-infected cells by phagocytosing virus-containing apoptotic bodies and dead cells (99). Neutrophils possess intracellular granules containing antimicrobial peptides, such as lactoferrin, defensins, cathelicidins LL-37, lysozyme, and neutrophil extracellular traps (NETs), comprising histones, decondensed chromatin, antimicrobial proteins, and proteases; these products are released to immobilize, inactivate, or kill MERS-CoV and thus inhibit further propagation (81). These cells are able to regulate both primary and secondary immune responses and release several chemokine molecules that can recruit further neutrophils to the site of infection for eradicating MERS-CoV.

*Airway NKCs* are an essential part of the innate immune response. They can be activated and proliferated within a few days in response to viral infection, such as MERS-CoV. These cells trigger cytotoxic activity and release substantial quantities of IFN-γ to consolidate the adaptive immune response, controlling and clearing virus-infected cells and inhibiting viral dissemination (21, 81). Besides cytokines with antiviral activity, NKCs secrete cytolytic granules that engage death receptors expressed on target cells to mediate cytolysis or contain perforin and granzymes that enter the target cell and trigger apoptosis through caspase-mediated signaling pathways. Moreover, NKCs, through their Fc receptors, play an important effector role in inducing ADCC, which involves an irreversible lytic change of MERS-CoV-infected cells recognized by specific antibodies (81). Overall, NKCs seem to serve several beneficial functions against MERS-CoV infection through supporting the immune response of cytotoxic T lymphocytes (CTLs), clearing viral infection, and facilitating cell growth and normal tissue repair (81, 100).

*Respiratory gamma-delta T cells* (*γδ T cells*) play the vital roles of initiating and regulating immune responses, reducing inflammation, and defending against viral infections, such as SARS-CoV and MERS-CoV, by alleviating severe lung damage, inhibiting pulmonary fibrosis, and enhancing tissue healing and repair (99, 101). In recovered patients with SARS-CoV, there were elevated numbers of memory γδ T cells’ populations that can produce IFN-γ markers to recruit additional immune effectors cells and, thereby, directly destroy SARS-CoV-infected cells and prevent patients to succumb due to SARS-CoV infection (81, 99, 102).

**Figure 3:**
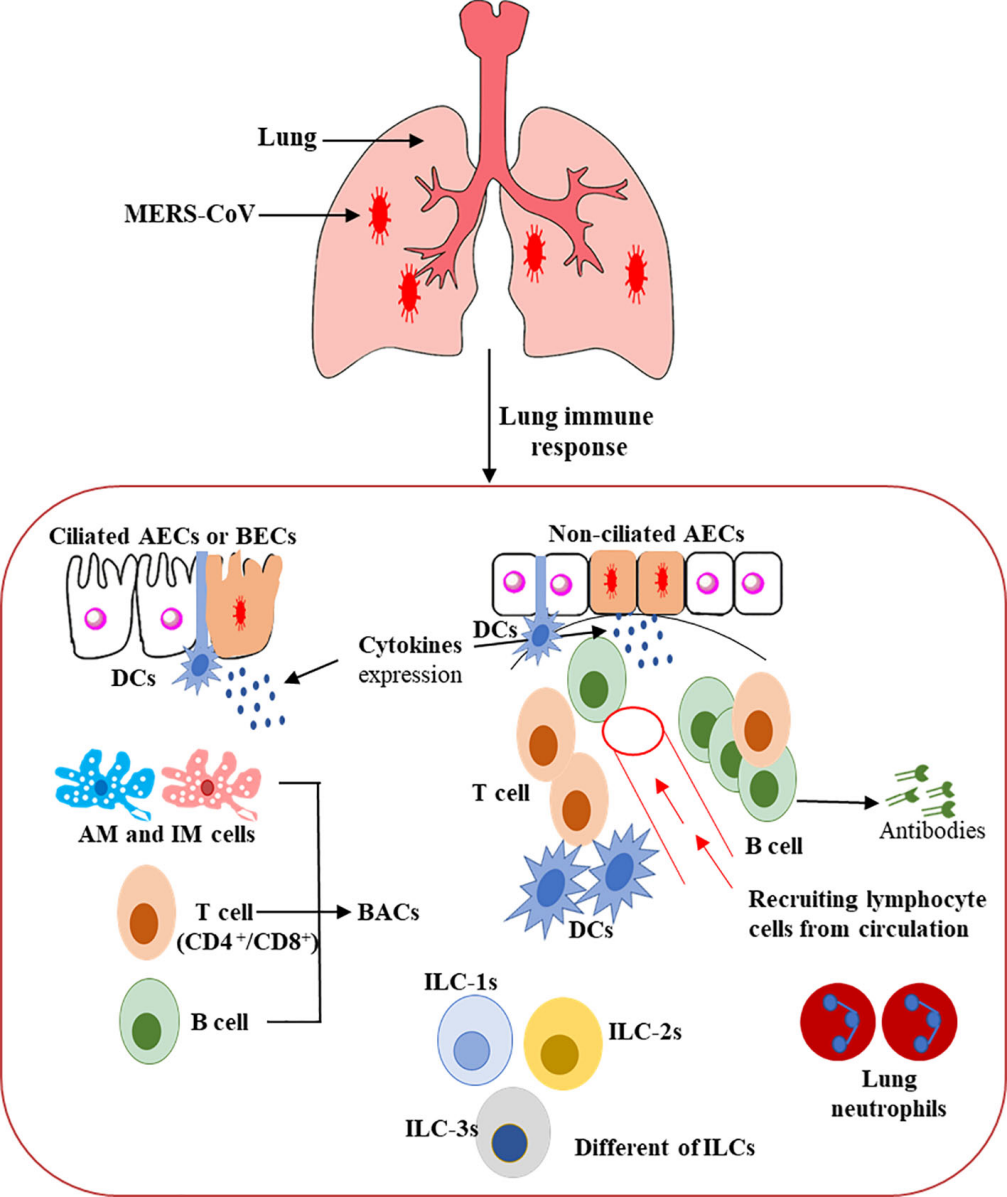
Schematic representation of the host immunity to MERS-CoV infection in the human lung (80–90). This figure shows the potential immunopathogenesis during MERS-CoV infection. Initially, host–viral entry was found at alveoli epithelial. The initial virus entry into the lung was observed in alveolar epithelia after binding of S glycoprotein RBD to the DPP4 receptor. The presence of the MERS-CoV particles leads to pro-inflammatory cytokine production from several ILCs, resulting in regulation of both innate and adaptive immune cells and accelerating the recruitment of immune effector cells at the site of infection for controlling and clearing the viral infection. CD4^+^ T cells and CD8^+^ cytotoxic T cells are recruited to the site of infection to kill virus-infected cells in the lungs. Activated B cells differentiate into plasma cells that can produce MERS-CoV-specific antibodies efficient in the lung not only by stopping the virus dissemination, but also by entering into blood circulation, thus conferring protection to other mucosal sites.

*DCs* are considered the fundamental orchestrator cells of the host immune system in the lung due to their capability to release numerous cytokine and chemokine molecules, activate T cells, and enhance defensive adaptive immune responses. They can be triggered indirectly, via the resident immune cells or respiratory epithelial cells that secrete various pro-inflammatory cytokine and chemokine markers, and directly by the invading virus, such as MERS-CoV, through PRRs (**Figure 3**) (103, 104). A balanced response by DCs that supports the effector cells’ responses to clear the virus without producing severe inflammation and lung tissue damage is possibly the key to controlling the virus infection or immune-mediated pulmonary damage (81, 104, 105).

The existence of viral infection, for example, MERS-CoV in the lungs, facilitates the migration of inflammatory monocytes from the blood circulation to the lungs with the assistance of the chemokine receptor, CCR-2. Monocytes yield pro-inflammatory cytokines, chiefly IFNs-I, and chemokines, and are also able to differentiate into DCs and macrophages, which enhance cytotoxic activity and T-cell activation and accelerate viral clearance (81, 106). The presence of inflammatory mediators in the lungs restricts the inflammatory responses and immune-mediated lung damage in addition to clearing viral particles of MERS-CoV (81, 106).

### 6.2 Adaptive immune responses in the lungs

The humoral and cellular adaptive immune response is critical for viral destruction, preventing viral replication and the production of newly infectious virions (107).

#### 6.2.1 Humoral immune response

Humoral immunity is critical for eliminating CoV infections, but little information is available about the underlying mechanisms (107). During viral infection of the LRT, plasma cells produce virus-specific antibodies that act directly to kill invading pathogens via several mechanisms, including neutralization, opsonization, inactivation of virion particles, and initiation of viral-infected cell clearing (107). The transmission of infectious virions from infected to non-infected cells must be inhibited to control virus spreading. Nabs efficiently bind to the surface proteins of infectious virions, preventing viral attachment to the target host’s cell receptors and subsequent entry (81). Nabs, although short-lasting, have been shown to elicit a protective response against the S glycoprotein of MERS-CoV in virus-infected cells (56, 61, 108). Corman et al. (109) found, in the analyzed serum samples, that over half of patients who died from MERS-CoV and all surviving patients developed IgG and Nabs responses against the virus. The existence of the MERS-CoV-specific antibodies plays a major role of eradicating the viral particles and in the development of memory B cells that that may be long-lasting, thus providing prolonged protection.

#### 6.2.2 Cellular-mediated immune responses: CD4^+^ T cells

The human LRT cellular immune response to MERS-CoV infection is not well-defined (110). Thus, whether CD4^+^ T cells, mainly Th-1 and Th-2 cells, can enhance the production of pro-inflammatory cytokines and chemokines in response to MERS-CoV infection remains uncertain (110). Few studies investigated the mechanisms of the T cells in response to MERS-CoV infection in animal models (52, 111). In response to virus infection, T cells, primarily CD4 cells, are important in facilitating the recruitment, proliferation, and differentiation of B cells into antibody-producing plasma cells or long-lasting memory cells in response to viral infection as well as for virus-infected cells’ elimination. The LRT memory CD4^+^ T cells can enhance robust protective responses against MERS-CoV and other CoV infections (81, 112, 113). The LRT memory CD4^+^ T cells, by targeting MERS-CoV and other CoVs, are able to produce cross-reactive, protective responses (81, 112, 114, 115). The LRT memory CD4^+^ T cells provide vital protection against a CoV challenge, as reported for SARS-CoV and MERS-CoV lung-infected transgenic mice (114). These protective immune responses are based on both IFN-γ secretion and the early, rapid initiation of strong innate and adaptive immune responses such as virus*-*specific CD8*^+^* T cells for the purpose of inhibiting viral replication (114). In addition, Zhao et al. (114) found that CD4^+^ and CD8^+^ T cells are able to persist and eliminate MERS-CoV-infected cells in the lung tissues of virus-infected BALB/c human leukocyte antigen (HLA)-DR2 and -DR3 transgenic mice.

#### 6.2.3 Cellular-mediated immune responses: CD8^+^ cytotoxic T cells

Cell-mediated immune responses, specifically involving CD8^+^ cytotoxic T cells, are essential for clearing intracellular pathogens (116). During respiratory viral infections, CD8^+^ cytotoxic T cells use numerous immune mechanisms to induce apoptosis and eliminate virus-infected cells in the lungs (**Figure 3**). CD8^+^ cytotoxic T cells directly bind to Fas death receptors (FasRs) on the cell surface of virus-infected cells and secrete cytotoxic granules, chiefly perforin and granzymes. These granules form pores in the membranes of target, virus-infected cells and induce intrinsic and extrinsic signaling apoptotic pathways. In SARS-CoV infection, for example, CD8^+^ cytotoxic T cells can control viral replication in infected cells and destroy pathogens (81). Zhao et al. (49), Zhao et al. (113), and Wang et al. (115) found that CD8^+^ cytotoxic T cells contribute to the elimination of lung-invading MERS-CoV virions in different animal models and provided permanent protection against subsequent infection. Another study, by Channappanavar et al. (52), illustrated that MERS-CoV-specific CD8^+^ T cells are also able to clear MERS-CoV virions in the lungs of both C57BL/6 and BALB/c mice that have been adenovirus 5 (Ad5) transduced with hDPP4 receptors to be susceptible to viral infection (52). The highest numbers of MERS-CoV-specific CD8^+^ T cells detected in the lungs of both hDPP4 transgenic C57BL/6 and BALB/c mice occurred 7 days post-infection (52). T cells, which are considered effective cellular immune responders, are the key defense of the immune system in combating and preventing respiratory virus infections (52, 81, 112).

## 7 Available MERS-CoV vaccines

The emergence of MERS-CoV has highlighted the urgent need for the development of effective vaccines, which are crucial for halting the spread of infection (117, 118). Although many MERS-CoV vaccine candidates are being investigated, none are currently licensed for human use. Several approaches for developing and evaluating MERS-CoV vaccines have been identified. These include the use of recombinant viral vectors, such as chimpanzee adenoviruses, adeno-associated viruses, MVA, pox-viruses, and measles viruses that can express a full-length S glycoprotein or an extracellular S1 domain and have been experimentally engineered, modified, and tested in animal models (119). These recombinant viral vector vaccines have been shown to induce an anti-S glycoprotein antibody response in addition to CD4^+^ and CD8^+^ cytotoxic T-cell responses in examined animal models (120). Based on safety and strong immunogenicity results, recombinant viral vector vaccines, including replication-deficient chimpanzee simian adenovirus vectors (ChAds) developed at Oxford University, are considered a promising human vaccine platform (121). The ChAdOx1-MERS-CoV vaccine contains the RBD of the MERS-CoV S glycoprotein (proteins stabilized trimer) that has been developed and evaluated in dromedary camels and mice, displaying promising outcomes, including excellent immunogenicity (a high titer of anti-MERS-CoV Nabs and robust CD8^+^ cytotoxic T-cell responses) and safety when encoding either adenovirus-human DPP4 (AdV‐hDPP4) or Rift Valley Fever viral (RVFV) S glycoproteins (122–124). Another study by Munster et al. (125) revealed that a single dose of the ChAdOx1-MERS-CoV vaccine could safely generate high IgG antibody levels and inhibit virus replication in the respiratory tract, decreasing the disease severity and providing protective immune responses in rhesus macaques. Thus, the ChAdOx1-MERS-CoV vaccine is well-tolerated, safe, and able to provoke both humoral and cellular responses in addition to inducing potent Nabs in mice and camel models (126). Another viral vector vaccine is the recombinant modified vaccinia virus Ankara (MVA) expressing the full-length MERS-CoV S glycoprotein (MVA-MERS-CoV S), and it is a stabilized trimer protein. This vaccine generated a substantial Nabs response in vaccinated BALB/c mice injected intramuscularly (i.m.) or subcutaneously (s.c.). In animal models, the MVA-MERS-CoV S vaccine also induced a specific IFN-producing CD8^+^ cytotoxic T-cell response against MERS-CoV infection through both routes (i.m. and s.c.) (127, 128). Based on the positive results obtained in the preclinical animal experiments, two human phase 1a and 1b clinical trials [Folegatti et al. (129) and Alharbi et al. (130)] were conducted to test the ChAdOx1-MERS-CoV vaccine, in healthy adults, aged between 18 and 50 from the Middle East and the United Kingdom. A single dose from the ChAdOx1-MERS-CoV vaccine was able to enhance both cellular and humoral immune responses against MERS-CoV. Therefore, the vaccine was deemed safe, well-tolerated, and highly immunogenic at all examined doses and has been moved forward to phase 2 human clinical trials for further evaluation (130). The ChAdOx1-novel coronavirus-19 (ChAdOx1-nCoV-19) vaccine has been granted an emergency use authorization during the coronavirus disease 2019 (COVID-19) pandemic. The vaccine showed high efficacy against infection and a very high level of protection against disease severity, hospitalization, and death (131, 132).

Recently, scientists developed another vaccine platform that utilizes genetic material termed messenger RNA (mRNA), which is introduced into the cell to express a viral protein for triggering the immune system (133). mRNA vaccines are encoding for a specific protein (e.g., RBD of S glycoprotein of CoV) and direct cells to produce copies of a desired protein of interest (e.g., the RBD of S glycoprotein of CoV) on their cell surface, allowing immune cells to recognize them and develop rapid immunity that protects against invasive pathogens. Currently, three mRNA vaccines for protection against COVID-19, the disease caused by SARS-CoV-2 infection, are approved for emergency use [e.g., Moderna, Janssen (Johnson & Johnson), and Pfizer-BioNTech]. In clinical trials, these vaccines have shown over 90% efficacy in preventing COVID-19 disease-related hospitalization. Both ChAdOx1-nCoV-19 and mRNA vaccines exhibit safety and high immunogenicity and are well-tolerated, with adequate and manageable reactogenicity in tested individuals (131, 132).

The MERS-CoV-RBD S glycoprotein-based subunit vaccine (a stabilized trimer protein) is another option, which is very effective, well-tolerated, and safe and can stimulate robust immune responses (134). The i.m. immunization of mice with a SARS-CoV-RBD protein-based vaccine showed long-term protection and could prevent viral replication in the infected animal (135). The MERS-CoV-RBD-based subunit vaccine is considered another potential strategy for controlling, managing, and preventing MERS-CoV infection. Thus, the subunit vaccine was capable of strong immunogenicity and of inducing, in vaccinated mice, high titers of Nabs that inhibit and block the binding of virus to target receptors and prevent the virus replication. This is a positive advance toward the development of efficient and safe MERS-CoV vaccines.

A strong neutralizing mucosal IgA antibody response against the RBD and MERS-CoV S glycoproteins was elicited by administering the MERS-CoV-RBD-based subunit vaccine intranasally (i.n.) (136). The i.n. administration induced a sound immune response in mice and generated Nabs in immunized rabbits that reduce the severity and lethality of the disease. Thus, i.n. vaccination is important to consider when developing an effective vaccine candidate that can eradicate MERS-CoV infection.

## 8 Potential adjuvants for the development of MERS-CoV vaccines

Immunizations with vaccines including specific adjuvants result in high Nabs production. Administration of MERS-CoV S glycoprotein together with an adjuvant resulted in a strong immune response and high levels of Nabs in vaccinated mice. When tested in animals, both alum and microfluoridized adjuvant 59 (alum- and MF59-containing adjuvants) when combined with MERS-CoV subunit vaccine induced cell-mediated and antigen-specific antibodies’ responses and protective immunity (137). However, another adjuvant, glucopyranosyl lipid A (a synthetic TLR-4 agonist), must be used in conjunction with alum to produce a strong cellular Th-1 immune response. These adjuvant–vaccine combinations will improve the strength and effectiveness of the MERS-CoV vaccines under development. Consequently, using proper adjuvants will play a key role in enhancing immunogenicity and safety as well as accelerating the development of a safe, well-tolerated, and effective MERS-CoV vaccine (137, 138).

Vaccines are one of the most effective measures to combat infectious disease, and as such, the ability to rapidly develop MERS-CoV vaccines is critical to public health. The considerable progress in establishing and designing a variety of vaccine platforms targeting MERS-CoV will play a key role in managing the infection (139). The development of effective and safe MERS-CoV vaccines has progressed, and some vaccine candidates have now reached human studies. These vaccines are based on viral vector platforms (ChAdOx1 and modified vaccinia Ankara [MVA]) and deoxyribonucleic acid (DNA) platforms (GLS-5300) that incorporate the MERS-CoV S glycoprotein antigen (140). The morbidity and mortality rates of MERS might be significantly reduced by an effective MERS-CoV vaccine. In order to address future MERS-CoV outbreaks, policymakers could implement a variety of approaches, one of which could be as straightforward as reactively vaccinating healthcare providers who are at high risk to contracting MERS-CoV infection during outbreaks (140). Preventing transmission of respiratory pathogens including MERS-CoV in hospitals and other places is important and requires the implementation of standard precautions of infection control procedures and protocols such as environmental and administrative controls in addition to personal protective equipment (PPE) and safer work practices (141). Effective intervention strategies have been established and are being maintained by public health agency in South Korea to combat MERS-CoV infection. These strategies include large-scale epidemiological research, rapid lab diagnosis, isolation, mass quarantine, social distancing combined with acquaintance quarantine, and clinical categorization of severe patients and treatment with suitable medical therapies (142). Currently, numerous therapeutic options have been used, such as intravenous immunoglobulin (IVIG), convalescent plasma (CP), whole blood therapy, monoclonal antibodies, and the repurposing of clinically approved treatments (143). These MERS therapies are urgently required due to the prerequisite for an efficient therapeutic approach to effectively block MERS-CoV S glycoprotein-mediated cell attachment, entry, and membrane fusion. Antimicrobial peptides (AMPs) are another potential alternative therapeutic agent for treating MERS-CoV infection because of their ability to inhibit viral protein–protein interactions (143). The development of potent antiviral vaccinations able to induce strong immune responses has been greatly facilitated by the developments of nanotechnology platforms. It has been demonstrated that antigen-specific activation of the humoral and cell-mediated immune responses is induced by antigen delivery via nanoparticle-based vaccines (144). Several nanoparticle vaccines such as liposomes, chitosan, and microspheres–nanoparticles have been developed and evaluated, and they show strong immunological responses in animal models (144). Several studies by Khan et al. [(139, 144, 145)] have demonstrated that Nabs, viral protease inhibitors, and interferons are considered crucial therapeutic approaches for the management of MERS-CoV infection. It has been proposed that liposomes and nanoparticles are active and potent vaccine adjuvants when tested in animal models (mostly mice) for biomedical research and vaccine efficacy studies (139, 144, 145). The encapsulated antigens can be delivered by liposomes to the APCs’ cytoplasmic compartment, where they are able to activate the immune system through cell-mediated mechanisms. It has been demonstrated that the liposomes and nanoparticle vaccines can induce robust cellular-mediated (e.g., CD4^+^ T cells, CD8^+^ T cells, DCs, and NKCs) and humoral (antibody-mediated) responses and increase the production of MERS-CoV-specific antibody in combating viral replication in examined mice (139, 144, 145).

## 9 Conclusion and further research prospects

Exploring the human mucosal immune responses and immunological mechanisms during MERS-CoV infection and vaccination in the URT and LRT during MERS-CoV infection and vaccination is an important task. Thus, in the current intensive review, we summarized and simplified the published studies investigating this topic. Innate immune cells, mainly APCs, such as macrophages, B cells, and DCs, mediate the immune response by recognizing PAMPs initiated by viral replication intermediates. These interactions lead to the rapid activation of antiviral signaling pathways and cytokine and chemokine production in response to the infection. T and B cells, the main two arms of the adaptive immunity system, play an important role in fighting MERS-CoV infection, particularly CD4^+^ helper T cells that regulate CD8^+^ cytotoxic T-cell and B-cell functions. CD8^+^ cytotoxic T cells are effector cells that directly eliminate virus-infected cells.

Notably, the human NALT plays a crucial role in protection against several respiratory pathogens due to a wide range of mucosal immune effector cells (146, 147). However, few data are available on the immunopathogenesis and immune responses during MERS-CoV infection in URT tissues because of the few reported cases worldwide. Thus, an urgent need exists to explore the role of cell-mediated immunity and the mechanisms of pro-inflammatory cytokines, chemokines, and cytotoxic markers in the mucosal tissues, mainly the tonsils, to better understand the immune response during MERS-CoV infection and vaccination.

The effective, efficient innate and adaptive immune responses in human lungs can play a crucial role in the immune response, combatting the respiratory viral infection, and prevention of further disease complications. Nevertheless, local immunity in lung tissues makes it difficult to understand and properly evaluate the immune responses following MERS-CoV infection or the benefits of human vaccine trials.

Future studies of immune responses in the URT and LRT following infection and vaccination are a priority, leading to the assessment of new vaccine formulations, doses, and routes of administration (e.g., i.n.). Finally, understanding in depth the mechanisms of mucosal immune responses in both the URT and LRT following MERS-CoV infection or administration of MERS-CoV vaccines could provide valuable information for developing preventive methods and therapeutic vaccine candidates that are able to stop, control, and manage current MERS-CoV infections or the emerging and re-emerging of other human CoV infectious diseases.
